# Uncovering culinary medicine research themes: Current status and future direction

**DOI:** 10.12688/f1000research.130947.1

**Published:** 2023-02-13

**Authors:** Jyothi Mallya, Thirugnanasambantham K, Pallavi Shettigar

**Affiliations:** 1 Library, Welcomgroup Graduate School of Hotel Administration, Manipal Academy of Higher Education, Manipal, Karnataka, 576104, India; 2 Food and Beverage Production, Welcomgroup Graduate School of Hotel Administration, Manipal Academy of Higher Education, Manipal, Karnataka, 576104, India; 3 Dietetics and Applied Nutrition, Welcomgroup Graduate School of Hotel Administration, Manipal Academy of Higher Education, Manipal, Karnataka, 576104, India

**Keywords:** culinary medicine, nutrition, gastronomy, research themes, knowledge assessment, impact measurement, acceptance and efficacy, implementation

## Abstract

**Background**: Culinary medicine (CM), an emerging discipline, is a novel approach that focuses on the art of food and cooking to prevent or improve health outcomes among chronic patients suffering from lifestyle diseases. The concept originated in the USA, gaining interest from scholars in medicine, nutrition, nursing, and the gastronomic discipline. Notably, in the last five years, there has been exponential growth in CM literature. In this regard, this study sought to examine the growth, performance and distinct research themes of CM literature over time.

**Methods:** To achieve the study’s objectives, this study employs descriptive, performance and bibliometric analysis. The descriptive analysis was applied to examine the growth of the CM literature since its emergence. The performance analysis was used to identify the most influential journals, articles, and authors in the CM domain. The bibliographic coupling analysis was adopted to discover the various research themes of the CM knowledge base.

**Results**: This study identifies three stages of literature development: Early stage, modest growth stage, and emerging stage. Further, the results indicate that most of the studies on CM had been conducted in developed countries. Our findings reveal a clear interest in integrating the CM curriculum into medical/nutrition education programs in recent years. Additionally, the study discovers four distinct main research themes: knowledge assessment, impact measurement, acceptance and efficacy, and implementation of CM.

**Conclusions**: These findings are helpful for scholars in medicine, nutrition, nursing, and gastronomy as they provide an overview of CM’s development and research focus. Future studies could focus on expanding the geographical distribution of research on CM and further exploring the identified research themes to gain a deeper understanding of the potential of this approach for improving health outcomes among chronic disease patients.

## Introduction

Lifestyle diseases kill almost 41 million people annually; around 71% of deaths globally ^1^ primarily result from lifestyle habits. Chronic diseases such as heart disease, stroke, diabetes, obesity, and some types of cancer are lifestyle diseases resulting from three modifiable lifestyle behaviors: smoking, eating poorly and being inactive. ^1^ According to a World Health Organization report, ^2^ chronic diseases accounted for 61% and 49% of all deaths and global disease burden, respectively. By 2030, it is projected that 56% of the world’s population will be afflicted with chronic diseases, accounting for 70% of all global deaths. However, healthier lifestyles and eating behaviors can prevent approximately 80% of chronic diseases because unhealthy dietary patterns are key drivers of inflammation and disease development. ^3^ A poor-quality diet has been linked to one in five deaths globally. ^4^^,^^5^ One of the fundamental rights of patients with chronic diseases is the timely administration of an appropriate diet to accelerate recovery and improve their quality of life. ^6^ It also underlines the necessity of offering an appropriate diet outside the hospital for the rapid recovery of chronic patients. ^7^

In his seminal text, ^8^ Brillat-Savarin defines Gastronomy as “the reasoned comprehension of everything connected with the nourishment of man” (1970, p. 52). He contends that gastronomy relates to history, physics, chemistry, cookery, commerce, and political economy and “[…] governs man’s whole life”. Later, other academics recognized the development of this discipline as crucial for sustainable and functional food. ^9^ Notably, the learning and training in gastronomy are also applied in the healthcare field to cultivate dietary recommendations for patients suffering from chronic diseases. ^10^ However, poor integration programs (For example, culinary nutrition, also known as culinary medicine) in medical curricula inhibit medical practitioners from playing the role of nutrition advisors and lifestyle models. ^11^

Culinary medicine (CM) is “a practice that incorporates the science of medicine and the art of food and cooking to create an individualized approach to food choices (p.1)”. ^12^ Regarding disease prevention and treatment, CM also considers social and cultural aspects of food consumption. ^13^ The primary purpose of CM is to spread awareness of the significant impact of food on health and disease. Additionally, it offers practical culinary training to impart knowledge on preparing foods with nutritional benefits to prevent, manage, and cure chronic diseases. ^3^ Robust evidence has shown that simple dietary changes can prevent or improve many chronic diseases. ^14^^–^^16^ Furthermore, a recent review study recommended the integration of nutrition competencies, such as skill-based nutrition training, into medical education. ^17^ Moreover, many medical schools in developed countries (USA, UK, and Australia) integrate the culinary medicine curriculum (CMC) into their clinical nutrition curriculum. ^3^^,^^18^

Over the last three decades, many studies on CM have suggested a growing interest among medical, social science, nursing, and gastronomy scholars. There were review ^19^ and scoping reviews evaluating the impact of nutritional interventions, ^20^ CM programs, ^13^ and CM interventions. ^21^ However, despite being a multidisciplinary and popular research field, there are no comprehensive bibliometric analyses of the CM knowledge base. Therefore, this study sought to answer three research questions. First, how has the CM knowledge base evolved since its beginning? Second, what are the most influential research constituents in the CM domain? Third, what are the research themes in the CM knowledge base? Our study also offers future research opportunities in the CM domain. Forty-seven documents from the Scopus database were analyzed to achieve these objectives. Specifically, the study findings will help future medical/nutrition/gastronomy education researchers identify challenges and opportunities in the CM domain. Our study findings are also helpful for CM chefs targeting chronic disease patients.

## Methods

### Data source, selection, and analyses

Data for this study were retrieved from the Scopus database. The journals indexed in Scopus were peer-reviewed. The indexing is based on four numerical quality measures: H-Index, CiteScore, SCImago journal rank and source normalized impact per paper. ^22^ Data for this study were selected using the keywords “culinary” and “medicine” in the article title. The search yielded a total of 69 records. Next, articles published in English journals were selected. Thus, a total of 47 documents were included. For the analyses, we used the VOSviewer software. This software is a freely available computer program developed to construct and view bibliometric maps. VOSviewer was used because of its more remarkable ability to represent the bibliographical map than other software visually. Specifically, VOSviewer helps to demonstrate large bibliometric maps that are easily interpretive. ^23^ Three types of analyses were conducted:1) descriptive analyses, 2) performance analyses, and 3) scientific mapping. Descriptive analyses deal with the growth of the CM literature and the subject-wise distribution of documents. We included the most influential research constituents in the performance analyses, such as journals, articles, and authors. Further, two scientific mapping analyses were conducted to uncover the research themes of the CM literature: Bibliographical Coupling Analysis (BCA).

## Results

**Figure 1.**
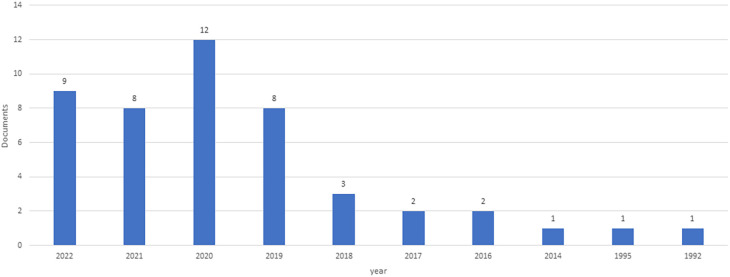
Growth of the literature year-wise.

Regarding the first question of the study, we outlined the advancement of the scientific production of the CM knowledge base. Figure 1 shows the development and trends, suggesting that the CM knowledge base is still emerging. Nevertheless, the growth rate over the years has not been equally dispersed. Between 1992-2016 (24 years), only 10% of the total contributions were published. Therefore, this period can be described as the “Early stage” of the CMs’ scientific production. During this period, there were early discussions on the importance of CM as a healthy eating habit. Between 2017-2019 (three years), there were 13 publications (28%). This period witnessed substantial growth in the number of publications. Thus, it can be described as a “modest growth stage” in the CM knowledge domain. During this period, a CMC was developed and integrated into the medical education curriculum to improve chronic disease management and prevention. Between 2020-2022, 29 articles were published. Thus, this period can be named as “Emerging stage” of the CM knowledge domain. Intervention studies were conducted during this period, and it was found that integrating CMC into medical and nutrition curricula affected students’ knowledge, attitudes, and behaviors.

### Country-wise contribution

**Figure 2.**
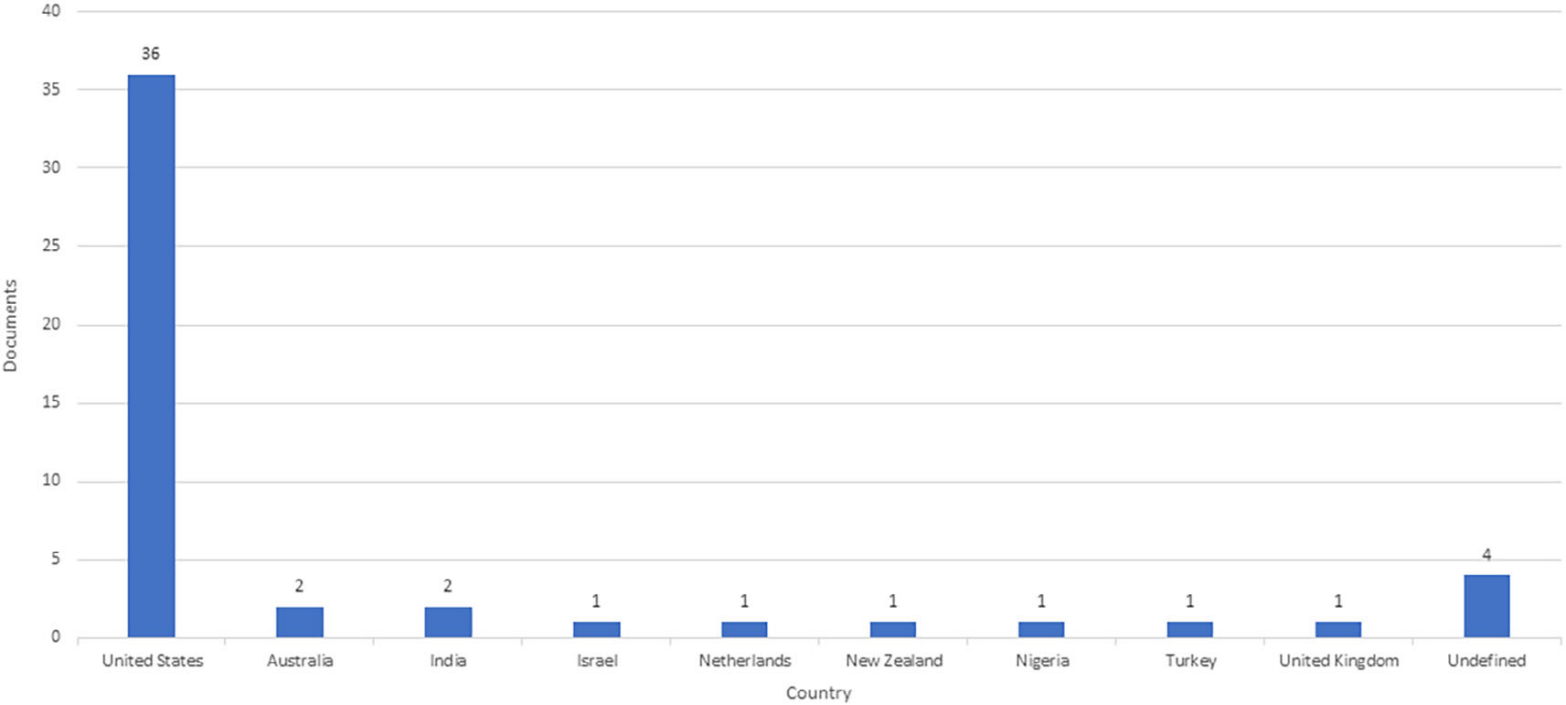
Country-wise distribution of documents.

A few countries involved in the scientific production of CM literature indicate that this domain is still not conceptualized in developing countries ( Figure 2). According to the following figure, 74% of the total scientific production is from the USA.

Our second query identified the most prominent constituents of the CM domain. Tables ( Tables 1– 3) represent the most influential journals, articles, and authors, respectively. While the count of publications denotes a journal’s productivity, the count of citations illustrates the academic influence and impact. ^24^ Therefore, based on the two-fold measures, the “American Journal of Lifestyle Medicine” was at the top, with 11 publications and 60 citations, followed by Medical Science Educator, with six documents and nine citations ( Table 1). However, in terms of citations, the articles published in the “Journal of Alternative and Complementary Medicine” and “Journal of Nutrition Education and Behaviour” had more citations than the journal “Medical Science Educator”. Notably, all these journals were in the Q1 or Q2 quartile of the Scopus journal index. This finding offered a comprehensive assessment of the journal quality of the CM studies. The results suggest that much of this field’s research meets a good quality standard. Further, it is also found that articles have been published in interdisciplinary subject domains such as medicine, nutrition, and alternative and complementary medicine.

**Table 1.** Journals with a minimum of two articles per journal.

No.	Journal name	Scopus percentile	Documents	Citations
1	American Journal of Lifestyle Medicine	61	11	60
2	Medical Science Educator	32	6	9
3	BMC Medical Education	82	2	8
4	Journal of Alternative and Complementary Medicine	75	2	28
5	Journal of Nutrition Education and Behaviour	66	2	20
6	Journal of The Academy of Nutrition and Dietetics	87	2	9

**Table 2.** Articles with a minimum of five citations.

No.	Author(s)	Title	Citations
1	Pang (2019)	Culinary medicine and community partnership: hands-on culinary skills training to empower medical students to provide patient-centred nutrition education	20
2	Ring (2019)	Cooking Up Health: A Novel Culinary Medicine and Service Learning Elective for Health Professional Students	17
3	Hauser (2019)	A Novel Culinary Medicine Course for Undergraduate Medical Education	14
4	Jaroudi (2018)	Impact of culinary medicine elective on medical students’ culinary knowledge and skills	12
5	Rothman (2020)	A Culinary Medicine Elective for Clinically Experienced Medical Students: A Pilot Study	11
6	Sicker (2020)	Implementing Culinary Medicine Training: Collaboratively Learning the Way Forward	8
7	Barkoukis (2019)	Culinary Medicine and the Registered Dietitian Nutritionist: Time for a Leadership Role	7
8	Hauser (2020)	The First, Comprehensive, Open-Source Culinary Medicine Curriculum for Health Professional Training Programs: A Global Reach	5

**Table 3.** Authors with a minimum of three articles with five citations.

No.	Author name	Documents	Citations
1	Dominique J. Monlezun	3	25
2	Alexander C. Razavi	3	25
3	Leah Sarris	3	25
4	John Wesley McWhorter	3	21
5	Tomy D. Dreibelbis	4	12
6	Daniel R. George	3	11

Article-wise citation resulted in eight documents with a minimum of five citations per document ( Table 2). The top eight articles are listed in Table 2. The article “Culinary medicine and community partnership: hands-on culinary skills training to empower medical students to provide patient-centred nutrition education” has had 20 citations since its publication in 2019. Therefore, it is safe to consider this article one of the seminal works in the CM domain. Next, we conduct an author-wise analysis. We identified six authors with at least three documents and five citations. Table 3 presents the six authors of this domain.

**Figure 3.**
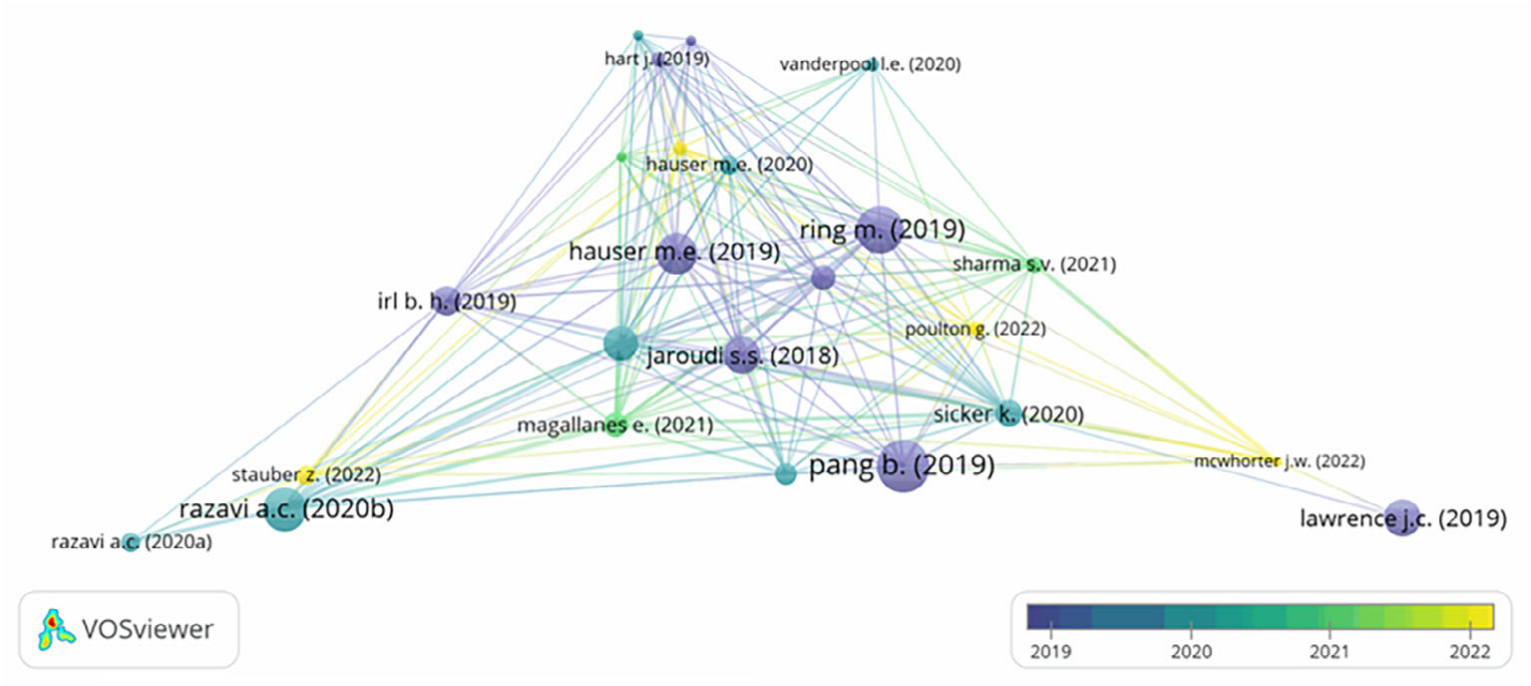
Network visualization map based on BCA.

The third query identified the intellectual structure and thematic trends of the CM knowledge base. For this purpose, we adopted bibliographical coupling analysis (BCA) using VOSviewer software. ^25^ Bibliographical coupling is the similarity of an article’s references. ^25^ In the BCA, the unit of analysis can be a document, source, or country. In our study, we used documents as the unit of analysis. Thus, BCA was conducted for articles with a minimum of two citations. This analysis resulted in 24 documents across the four clusters. Figure 3 shows the results of the analysis. A larger circle represents a superior connotation for a document. ^26^

### Cluster one: Knowledge assessment

Cluster one has eight articles. Of these, three articles were commentaries on providing a robust CM for health professionals to support patients’ health outcomes, ^27^ home cooking prescriptions by clinicians, ^28^ and the mechanism of how food and beverages work in the body. ^29^ The remaining four articles assessed participants’ pre- and post-nutrition knowledge, attitudes, and counselling confidence. ^30^^–^^33^ The findings of these studies provide empirical evidence for significant improvement in knowledge, attitude, and healthy cooking behaviors among the respondents. However, scholars recommend further inclusion of resources in CM courses to meet participants’ demands. ^30^ Most of the studies in the clusters have adopted the questionnaire method to assess respondents’ pre- and post-nutrition knowledge, attitudes, and counselling confidence. ^30^^,^^32^^,^^34^ The last document was a Master’s thesis ^34^ that assessed first-year medical students’ knowledge, attitudes, behaviors, and confidence based on a mixed-method research approach.

### Cluster two: Impact measurement

Cluster two contained six articles. Studies in this cluster have assessed the impact of CM education on lifestyle medicine counselling, ^35^ existing nutrition intervention, ^36^ counselling confidence, ^5^ dietary knowledge and behaviours, ^35^ and outcome of CM education. ^37^ The findings suggest that hands-on cooking-based nutrition education, such as Cooking for Health Optimization with Patients (CHOP), significantly impacts Mediterranean diet intake more than traditional nutrition education versus traditional curricula. ^35^ Additionally, participants participating in the CM program were more confident discussing nutrition intake with their patients. ^5^ More importantly, hands-on culinary education courses were positively associated with adherence to the Mediterranean diet and improved knowledge of healthy eating. ^35^ Thus, such interventions are cost-effective for addressing lifestyle diseases, such as obesity and chronic illness. ^35^ Specifically, a study conducted among 1031 Heart failure (HF) admissions found that medication and dietary noncompliance were two significant contributing factors for readmissions (34.62% and 16.92%, respectively). Other factors included comorbidity, HF exacerbation, iatrogenic factors, and drug abuse. In contrast to conventional care, providing CM education to patients with HF would have avoided 93 HF readmissions and saved $3.9 million over an expected 4-year timeframe. ^38^

### Cluster three: Acceptance and efficacy

In cluster three, there are six studies. Of six, four studies measure the impact of the CM course on nutrition. ^39^^–^^42^ The other two studies were commentary papers on integration ^43^ and CM’s importance of CM ^44^ in the medical curriculum. The findings of the studies included in this cluster suggest that the CM elective is highly acceptable ^40^ and valuable to the medical school curriculum. ^42^ These findings also provide evidence for the successful integration of the CM elective course in the nutrition program to combat the rising rates of obesity, diabetes, and preventable diseases related to nutrition. ^40^

### Cluster four: Implementation of CM program

Cluster four comprised four articles. The studies in this cluster discuss the implementation of CM training, ^45^ the integration of CMC as an interprofessional competency, ^46^ the impact of CMC on biometric and psychological factors, ^47^ and barriers to implementing CMC. ^48^ Studies have adopted research approaches, such as intervention, ^49^ quasi-experimental design, ^47^ and focus groups. ^48^ An experimental study ^47^ also established a substantial decline in HbA1c levels among participants. Furthermore, this study demonstrated a significant increase in the consumption of fruits and vegetables. In other words, the findings of this study suggest a potentially positive impact on health outcomes among patients with type 2 diabetes. ^47^

## Discussion and recommendations for future studies

This first bibliometric study aimed to advance understanding of current research trends and streams in the CM knowledge domain. Our findings suggest a clear interest in integrating CMC into medical/nutrition education programs in recent years. In 2022, 14 new publications evaluated the viability of using CMC in medical/nutritional education. ^13^^,^^21^^,^^41^^,^^50^^–^^52^ Studies have also evaluated knowledge and confidence in counselling. ^31^^,^^35^

Based on the years of growth of the CM literature, we have identified three stages of literature development: Early stage (1992-2016), modest growth stage (2017-2019) and emerging stage (2020-2022). During the early stage, studies focused mainly on understanding the culinary metaphor. ^53^^,^^54^ During the modest growth (2017-2020), most studies were on the feasibility, training, integration, and efficacy of CMC. ^28^^,^^30^^,^^33^^,^^36^^,^^39^^,^^40^^,^^42^^,^^43^^,^^49^^,^^55^^–^^57^ A study on formulating a natural medicine based on three culinary herbs for a skin infection ^58^ was also published during the early stage. However, the emerging stage (2020-2022) witnessed prolific growth in literature. Of 47, 29 articles were published during this period. Researchers advanced the CM knowledge base by studying the impact of CM education, ^50^^,^^51^^,^^59^ barriers to implementing CMC, ^48^^,^^60^ remote learning of CM, ^41^^,^^47^^,^^52^ and the impact of CM training on patients’ health. ^61^ Our findings also revealed that most of these studies had been published over the last five years. Of 47, 40 were published between 2018-2022. Studies have also achieved statistically significant outcomes regarding the promotion of hands-on CM methods over traditional didactic methods to train and assist chronic patients as future physicians.

Our findings also revealed that most published studies were from the United States of America (36 out of 47). This outcome suggests that increasing interest in CM is geographically concentrated and limited. However, this emphasis on North America suggests the need for greater international participation to validate CMC as an effective strategy for nutrition instruction in medical education. It also emphasizes the significance of considering cultural relevance and the probable need for adjustment when implementing international norms. ^21^ CM is an evidence-based field that blends the art of food and cooking with the science of medicine. ^29^ It is considered to help individuals make personal medical decisions regarding healthy eating habits, which helps them to prevent lifestyle diseases and restore well-being. Furthermore, CM attempts to improve a patient’s condition with what she or he regularly eats and drinks. ^12^ Therefore, we recommend that researchers in developing countries focus on integrating CM programs into medical/nutrition programs to benefit individuals’ overall well-being.

Our findings also revealed that most articles were published in medical journals. A healthy diet is one of the four factors that appear to be linked to an 80% risk reduction in deadly chronic diseases. ^1^ In 2020, the World Health Assembly called for developing a national public health policy framework to establish programs for the prevention and control of chronic diseases, emphasizing developing countries. However, there is minimal public awareness regarding the association between health and lifestyle. Modest but achievable lifestyle changes are likely to considerably impact individual and population levels. Therefore, we recommend that future studies in the CM domain focus on these aspects.

The performance analyses of research constituents identified the most prolific journals, articles, and authors. The “American Journal of Lifestyle Medicine” emerged as the most influential journal, with 11 documents and 60 citations. The second most influential journals in documents and citations were “Medical Science Educator” and “Journal of Alternative and Complementary Medicine”. Further, the two documents from the “Journal of Nutrition Education and Behaviour” had 20 citations. This indicates that the CM is an interdisciplinary discipline.

Given the recency of this domain, our study identified four major research themes in the CM domain. The identified domains were knowledge assessment, impact measurement, acceptance and efficacy, and CMC implementation. There is a lack of studies on program costs. ^21^ The additional costs involved in personnel, facilities, consumables, and types of equipment remain understudied. Furthermore, these costs may differ significantly between programmes and locations. ^21^ Moreover, although food prescription and CM programs are gaining popularity, there are not enough studies on how physicians/nutritionists can provide evidence-based nutritional information tailored for culturally diverse low-income populations. ^62^ Therefore, this could be another area of research interest in designing a CM program targeted at low-income, food-insecure people. Therefore, we recommend that future studies address this gap.

CM’s beneficial role in improving health and reducing healthcare costs must be integrated beyond medical/nutritional education. ^3^ Focusing more on healthy eating habits and understanding the nuances of the scientific cooking method is critical to reducing chronic disease burden and healthcare expenses. Empowering and teaching individuals about healthy eating habits is cheaper than prescriptions and aggressive treatments to manage chronic diseases. ^3^ Therefore, we recommend that future research should focus on this literature gap. The integration and efficacy of CM programs should be conducted with a different set of samples, such as public and community health professionals. Similarly, although medical/nutrition education professionals are well-trained to deliver nutrition education to students, this could also be offered to school communities. ^63^

Gastronomy, a new interdisciplinary approach to food, has been recognized as a vital yet underexplored area of academia. ^9^ In developed countries, such as the USA and the UK, CM chefs prepare a diet for chronic patients. These CM chefs prepare food based on recipes provided by dietitians. ^64^ Furthermore, because of the popularity of healthy nutrition, culinary services with special menus, such as gluten-free, are extended to chronic patients by chefs without sufficient knowledge. ^3^ Previous studies also revealed that a vegan diet is associated with a lower risk of diabetes, ^65^ CVD, and IHD. ^66^ However, our study did not find any studies on other popular diets, such as the vegetarian diet, and their impact on chronic patients. This gap can be addressed by the collaborative efforts of gastronomists ^12^ and dietitians to improve performance in the health sector. Therefore, we recommend that future studies in the domain of CM focus on these research gaps in the literature. Future studies should also consider integrating vegetarians and other popular diets into CMC. The findings of these studies can offer concrete and robust ways for healthcare solutions to improve the quality of life of chronic patients.

### Implications

This study adds to the body of the CM knowledge base in several ways. This study was the first to demonstrate the thematic evolution of critical research themes in the CM domain. By doing so, this study advances CM’s learning and comprehension of CM. This paper also explains the future direction of this field of study. Second, this study advances the field’s theoretical advancement by assisting researchers in identifying prospective avenues for CM. Given recent findings, this study encourages medical/nutritional educators to innovate and integrate CM initiatives into medical/nutritional education. This study identified a subtle but noteworthy body of literature describing the evolution of the CM knowledge base over time since its emergence. This study also empirically identified the most influential research constituents in the CM domain. Our study also adds to the body of knowledge by scientifically identifying different clusters in the CM domain. The four clusters that emerged were *knowledge assessment, Impact Measurement, acceptance and efficacy, and implementation* of the CM program. Our study is helpful for future researchers in the domain of CM, as they can visualize the evolution and development of the CM knowledge base concerning the assessment, measurement, acceptance, and implementation of the CM. Further, our study is valuable to practitioners and policymakers who wish to implement, measure, and assess CM programs.

### Limitations

Irrespective of its contribution to the body of CM knowledge, this study has a few limitations. First, the documents for this study were included only from the Scopus database. Though the Scopus database is one of the reputed databases, the studies from the other databases could enrich the study. Therefore, we recommend that papers from other databases be used in future research. Second, this study only included articles in English. Thus, it is advised that future studies should include non-English articles. Third, software such as VOS viewer places the same threshold value for documents at different points when performing bibliometric analysis.

## Conclusion

For the last five years, there has been a scholarly interest in CM among scholars across different domains of literature. Many medical schools have successfully integrated CMC into their programs in developed countries, such as the USA and Australia. However, developing countries are yet to attempt to include CMC in their programs. The inclusion of CMC is essential for several reasons. First, teaching culinary skills to children and youth will likely prevent lifestyle diseases such as obesity, diabetes, and heart disease. Second, culinary skills and competencies are associated with an increased intake of fruits and vegetables. Third, most studies included in this review found that culinary skills in medical/nutrition education enhance knowledge, attitude, self-efficacy, and health-promoting behaviours among physicians and dieticians. Fourth, CMC offers a unique opportunity for experiential learning, further encouraging nutrition-led behaviour among the youth. Most importantly, CM interventions appear logical in targeting chronic diseases because unhealthy eating habits are considered attributing factors, such as lifestyle diseases. Fifth, properly integrating CMC in medicine and nutrition curricula helps reduce healthcare costs. Finally, given the significance of healthy cooking and eating habits in preventing chronic diseases, if adopted scientifically and successfully, CM plays a vital role in overall well-being and reduction in healthcare costs. Capitalizing CM as a tool to improve dietary patterns is the most significant step in incorporating lifestyle into medicine. To be successful in community outreach efforts, enhanced CM education is imparted in medical, nutritional, and culinary programs. CM programs should be expanded and promoted in developing countries. This expansion and inclusion may further impact chronic patients and overall community well-being.

## Author contributions

Ms Pallavi Mahesh Shettigar: conceptualization, writing - original draft; writing - review & editing, validation

Dr Chef K. Thirugnanasambantham: conceptualization, methodology, resources, supervision, validation, visualization

Ms Jyothi Mallya: conceptualization, data curation, formal analysis, methodology, software, writing the original draft

## Data Availability

Figshare. Culinary Medicine. DOI: https://doi.org/10.6084/m9.figshare.21973925.v1. ^67^ This project contains the following data:
This is bibliographic data of articles on culinary medicine Data are available under the terms of the Creative Commons Attribution 4.0 International license (CC-BY 4.0).
