# Supplementary material for: Uncovering culinary medicine research themes: Current status and future direction
Source: F1000Res. 2023 Feb 13;12:173. [Version 1] doi: 10.12688/f1000research.130947.1 (PMC10696491; doi:10.12688/f1000research.130947.1)
Supplement: Peer review history

# Reviewer report: Reviewer response for version 1

**DOI:** 10.5256/f1000research.143746.r215298

**Published:** 2023-11-30

**Authors:** Sanjeev Kumar Saxena

**Affiliations:** 1 Assam University, Silchar, Assam, India

The text highlights the global impact of lifestyle diseases, attributing approximately 71% of deaths to modifiable behaviors such as smoking, poor diet, and inactivity. It introduces the concept of Gastronomy and emphasizes the integration of culinary medicine (CM) into healthcare for preventing and managing chronic diseases through personalized dietary approaches. The study conducts a bibliometric analysis, revealing the evolution of CM literature in three stages and the dominance of the United States in scientific production. It identifies major research themes, including knowledge assessment, impact measurement, acceptance and efficacy, and CM program implementation. The study underscores the need for future research to address gaps in program costs, the impact of CM on diverse populations, and the integration of popular diets into CM. Overall, the findings advocate for the expansion of CM programs, particularly in developing countries, to positively impact chronic patients and community well-being.

Is the work clearly and accurately presented and does it cite the current literature?

Yes

If applicable, is the statistical analysis and its interpretation appropriate?

I cannot comment. A qualified statistician is required.

Are all the source data underlying the results available to ensure full reproducibility?

Yes

Is the study design appropriate and is the work technically sound?

Yes

Are the conclusions drawn adequately supported by the results?

Yes

Are sufficient details of methods and analysis provided to allow replication by others?

Yes

Reviewer Expertise:

Hospitality & Tourism Management

I confirm that I have read this submission and believe that I have an appropriate level of expertise to confirm that it is of an acceptable scientific standard.

# Reviewer report: Reviewer response for version 1

**DOI:** 10.5256/f1000research.143746.r200890

**Published:** 2023-11-15

**Authors:** Ashish Dahiya

**Affiliations:** 1 Maharshi Dayanand University Rohtak, Rohtak, Haryana, India

**Brief:** "Uncovering Culinary Medicine Research Themes: Current Status and Future Direction" is a well-presented and informative article that contributes to the understanding of the growth and research themes in culinary medicine literature. While it effectively supports its conclusions with data, providing more information about the sources of data would enhance the study's reproducibility. Overall, it is a valuable contribution to the field of culinary medicine and offers insights for future research directions. **However, there is a question which must be answered that India has been observing Culinary Medicine through Ayurveda. This part is silent in the article. Similar contexts can also be observed in other parts of world.**

**Detailed Report:** The article titled "Uncovering Culinary Medicine Research Themes: Current Status and Future Direction" exhibits a well-organized structure and effectively communicates its research findings. It adeptly introduces the background, objectives, methods, and outcomes of the study, offering a comprehensive understanding of the subject matter. The article properly integrates current literature, discussing the emergence and significant growth of culinary medicine (CM) as a discipline over the past five years. This effectively contextualizes the research.

 The study's design aligns appropriately with its research objectives. It employs descriptive, performance, and bibliometric analyses to scrutinize the growth, performance, and research themes within CM literature, which are technically sound approaches for fulfilling the study's aims. Descriptive analysis tracks CM literature's growth, performance analysis pinpoints influential journals, articles, and authors, and bibliographic coupling analysis unveils key research themes. These combined methods provide a holistic overview of CM's development.

 The article generously offers methodological details, allowing readers to comprehend and potentially replicate the study. However, more information regarding specific bibliometric metrics and thresholds would bolster the methodological transparency.

 The study heavily leans on bibliometric analysis, focusing on publication and citation data. While traditional statistical analysis is not applied, this method remains appropriate for discerning trends and research themes within CM literature. The interpretation of the findings is robust, successfully identifying research themes and stages of CM literature development.

 The article does not explicitly address the availability of the source data underpinning the results, an aspect that is typically emphasized in bibliometric studies. Including references to the databases and data sources used for collection would enhance reproducibility.

 The conclusions drawn are well substantiated by the results, with the study effectively identifying three stages of CM literature development, the geographic distribution of research, and four distinct research themes. These conclusions align with the presented data, offering valuable insights for scholars in various fields. The call for future research to explore CM's potential for enhancing health outcomes among individuals with chronic diseases is logically founded on the study's findings.

Is the work clearly and accurately presented and does it cite the current literature?

Yes

If applicable, is the statistical analysis and its interpretation appropriate?

Yes

Are all the source data underlying the results available to ensure full reproducibility?

Yes

Is the study design appropriate and is the work technically sound?

Yes

Are the conclusions drawn adequately supported by the results?

Yes

Are sufficient details of methods and analysis provided to allow replication by others?

Yes

Reviewer Expertise:

Hospitality & Tourism Education, Culinary Research, Indian Food, Hotel Operations, Housekeeping

I confirm that I have read this submission and believe that I have an appropriate level of expertise to confirm that it is of an acceptable scientific standard.
